# Understanding the Role of Mucosal-Associated Invariant T-Cells in Non-human Primate Models of HIV Infection

**DOI:** 10.3389/fimmu.2020.02038

**Published:** 2020-08-18

**Authors:** Isaac M. Barber-Axthelm, Stephen J. Kent, Jennifer A. Juno

**Affiliations:** ^1^Department of Microbiology and Immunology, The Peter Doherty Institute for Infection and Immunity, The University of Melbourne, Melbourne, VIC, Australia; ^2^Department of Infectious Diseases, Melbourne Sexual Health Centre, Alfred Hospital and Central Clinical School, Monash University, Melbourne, VIC, Australia; ^3^ARC Centre for Excellence in Convergent Bio-Nano Science and Technology, The University of Melbourne, Melbourne, VIC, Australia

**Keywords:** MAIT cells, MR1, non-human primate, comparative immunology, HIV/SIV infection, HIV/TB co-infection

## Abstract

Chronic HIV infection causes systemic immune activation and dysregulation, resulting in the impairment of most T-cell subsets including MAIT cells. Multiple human cohort studies demonstrate MAIT cells are selectively depleted in the peripheral blood and lymphoid tissues during HIV infection, with incomplete restoration during suppressive antiretroviral therapy. Because MAIT cells play an important role in mucosal defense against a wide array of pathogens, fully reconstituting the MAIT cell compartment in ART-treated populations could improve immunity against co-infections. Non-human primates (NHPs) are a valuable, well-described animal model for HIV infection in humans. NHPs also maintain MAIT cell frequencies more comparable to humans, compared to other common animal models, and provide a unique opportunity to study MAIT cells in the circulation and mucosal tissues in a longitudinal manner. Only recently, however, have NHP MAIT cells been thoroughly characterized using macaque-specific MR1 tetramer reagents. Here we review the similarities and differences between MAIT cells in humans and NHPs as well as the impact of SIV/SHIV infection on MAIT cells and the potential implications for future research.

## Introduction

Unconventional T-cells, including mucosal-associated invariant T-cells (MAIT cells), have emerged as important immune mediators in both infectious and inflammatory diseases [Reviewed in Godfrey et al. (1)]. While their role in cancer development and progression has not been fully elucidated, MAIT cells and specific populations of major histocompatibility complex class I-related (MR1)-restricted T-cells have been shown to infiltrate the tumor microenvironment and are able to lyse cancer cells *in vitro* and *in vivo*, indicating a potential role in cancer immunotherapy (2); Reviewed in Lukasik et al. (3). In contrast to conventional T-cells which recognize antigens through MHC-mediated antigen presentation, MAIT cells recognize antigens by MR1-mediated presentation of vitamin B metabolites predominately through a Vα7.2 semi-invariant T-cell receptor (TCR) (4, 5). MAIT cells are also capable of being activated via a cytokine-mediated, antigen independent pathway, indicating they have properties of both innate and adaptive immune cells (6). These features of MAIT cells highlight their importance in rapid immune responses to a wide array of pathogens.

While the ability of MAIT cells to mount an immune response against bacterial pathogens is well established, their role in viral infection is not as clear. Viral replication cycles do not involve vitamin B synthesis pathways, which are necessary for TCR-dependent MAIT cell activation. Multiple studies have shown increased MAIT cell activation in patients with active viral infections, including dengue (7, 8), influenza (7), chronic hepatitis B (9), hepatitis C (7), and Severe Acute Respiratory Syndrome coronavirus 2 (SARS-CoV-2) (10). Similar findings have also been made in mice following influenza infection (11), as well as *in vitro* with hepatitis C (7), influenza (12), and zika virus (8). Virus-induced MAIT cell activation is mediated through TCR-independent pathways, as shown for influenza (7, 12), dengue (7), hepatitis C (7), or zika virus (8) exposure *in vitro*. This stands in contrast to MAIT cell stimulation with paraformaldehyde fixed *E. coli*, which is blocked by treatment with an anti-MR1 blocking antibody. Beyond MAIT cell activation, data on MAIT cell contributions to viral immunity is limited. Supernatant from activated MAIT cells suppresses hepatitis C virus replication in a dose-dependent manner (7). MR1^–/–^ mice, which are deficient in MAIT cells, have increased morbidity and mortality following influenza challenge; compared to wild type controls (11). Increased morbidity and mortality is ameliorated with adoptive transfer of murine pulmonary MAIT cells prior to viral challenge. Adoptive transfer of pulmonary MAIT cells also reduced morbidity and mortality in Rag^–/–^ IL-2Rγ^–/–^ mice following influenza challenge (11). Taken together, these studies indicate that MAIT cells are capable of suppressing viral replication and providing clinical improvements during viral infections against certain viruses. Additional studies are needed to better characterize the impact MAIT cells have, both beneficial and deleterious, on viral infections, as well as the breadth of viral infections that MAIT cells are able to mount an immune response against.

A better understanding of MAIT cell function will require continued evaluation of human MAIT cell dynamics during various disease states, along with *in vivo* models that best represent the relevant pathologic processes. The MR1 gene, the primary receptor for MAIT activation through antigen presentation, is highly conserved among mammalian species, but is absent in non-mammalian species (13). Additionally, there have been 3 separate losses of functional MR1 among mammals, including in Lagamorpha (rabbits), and in Carnivora (dogs, cats, and ferrets) (14). Mice carry a functional MR1 gene but have a relatively low abundance of MAIT cells in the peripheral blood (median: 0.1%) necessitating the generation of transgenic mice expressing an invariant mVα19-Jα33 TCRα to increase MAIT cell frequencies (15, 16), or the boosting of tissue MAIT cell frequencies by administration of antigen and TLR agonists (17). In contrast, non-human primates (NHPs) express a functional MR1 gene and maintain MAIT cells at frequencies more comparable to humans, providing a superior *in vivo* model to study MAIT cell immunological dynamics. Herein, we discuss the current state of MAIT cell characterization in NHPs [which has focused on rhesus macaques (RM), pigtail macaques (PTM), and Mauritian cynomolgus macaques (MCM)] and the changes in MAIT cell populations that occur during simian immunodeficiency virus (SIV) and simian-human immunodeficiency virus (SHIV) infection, which are the critical animal models for HIV infection.

## Phenotype of Non-Human Primate Mait Cells

Human MAIT cells were originally identified as Vα7.2^+^ CD161^+^ cells among the bulk T-cell population [Reviewed in Garner et al. (18)]. Recently, the development of antigen loaded MR1 tetramers has allowed for a more refined identification of MAIT cells by flow cytometry (19, 20). Similar approaches have been utilized to phenotype macaque MAIT cells, via identification of Vα7.2^+^ and/or MR1-5-OP-RU^+^ T-cells (21–29). One important consideration for determining tetramer reactivity in macaque MAIT cells is the utilization of species specific MR1 tetramers. Two studies have identified incomplete cross reactivity of human MR1 tetramers with macaque MAIT cells (23, 25). Identification of these cells is improved with the use of macaque specific MR1 tetramers. Furthermore, the inclusion or exclusion of Vα7.2 expression in the definition of a MAIT cell should be carefully considered. There is growing evidence of a unique Vα7.2^–^MR1 tetramer^+^ T-cell population in humans (30, 31), which has also been identified in the peripheral blood of PTMs, RMs, and MCMs (23, 25, 28). Additional work is needed to characterize these cells and to compare their phenotypic and functional properties to their human counterparts.

Human MAIT cells are predominately CD8αα^+^ or CD4^–^CD8^–^, with a minor population of CD4-expressing cells (19). In contrast, NHP MAIT cells are almost uniformly CD8α^+^, with 3 studies noting an absence of CD4^–^CD8^–^ MAIT cells in NHP (23, 25, 28). One additional study identified peripheral blood MAIT cells as predominately CD8α^+^ (36.3%) or CD8^–^CD4^–^ (44.9%) in RMs, with minor populations of CD8^+^CD4^+^ (2.9%) and CD4^+^ (15.8%) MAIT cells (29). MAIT cells were identified based on reactivity to NHP-specific MR1 tetramers without concurrent expression of Vα7.2, which may partially explain the presence of CD8^–^CD4^–^, CD8^+^CD4^+,^ CD4^+^ MAIT cells that were not observed in other studies. It is presently unknown if NHP CD8α^+^ MAIT cells express a homodimeric (CD8αα^+^) or heterodimeric (CD8αβ^+^) receptor. The cause for this absence of CD4^–^CD8^–^ MAIT cells in the majority of NHP studies is unknown, and additional studies are needed to characterize this variation from human peripheral MAIT cells. Human CD8^+^ and CD4^–^CD8^–^ MAIT cells have been shown to have distinct phenotypic and functional profiles (32). CD8^+^ MAIT cells express higher levels of cytotoxic and coactivating markers compared to CD4^–^CD8^–^ MAIT cells, and produce higher levels of IFNγ and TNFα following stimulation. CD4^–^CD8^–^ MAIT cell can be derived *in vitro* from CD8^+^ MAIT cells following TCR-dependent activation. Potential causes for the relative paucity of CD8^+^ MAIT cells in captive NHPs include species-specific variation in MAIT cell development or differentiation between humans and NHPs, or environmental factors related to husbandry practices which drives the altered frequencies in NHP peripheral CD8^+^ and CD4^–^CD8^–^ MAIT cells. A lack of CD4^–^CD8^–^ MAIT cells in NHPs may also impact the immune response to certain disease states, and should be considered when utilizing NHPs as a model for humans. While NHPs predominately lack CD4^–^CD8^–^ MAIT cells, we caution against pre-gating on CD8^+^ T cells prior to identifying MR1 tetramer^+^ cells in NHPs, as this could hinder observing shifts in MAIT cell CD4 and CD8 co-receptor usage during disease states or experimental interventions.

CD161, a C-type lectin like-receptor, is almost ubiquitously expressed by human MAIT cells in the peripheral blood and has classically been used as a co-staining marker to gate on Vα7.2^+^ or MR1 tetramer^+^ cells (15, 33, 34)This marker is associated with innate-like, TCR-independent responses to IL-12 + IL-18 stimulation (35, 36). Three studies have utilized CD161 expression to identify MAIT cells in NHPs (22, 26, 27). However, two studies have noted poor cross-reactivity of anti-human CD161 antibodies against RM and PTM CD161 antigen (23, 25). Additionally, CD161 downregulation has been identified in MAIT cells from chronic HIV infected individuals (33, 37, 38). As a result, we strongly caution against the use of CD161 to define the total MAIT cell population in NHPs; rather, MR1 tetramer in combination with TCR Vα7.2 can distinguish major MR1-restricted T-cell populations, which can then be phenotyped for additional surface markers. PLZF (ZBTB16) is most consistently expressed on MAIT cells in NHPs (21, 23–25, 27, 28), as well as in humans (34, 39), and is a marker to consider when identifying the bulk MAIT cell population. Additional markers that are expressed on a relatively high frequency of MAIT cells and may be considered for MAIT cell identification are CCR6 and IL-18Rα, which have been shown to be expressed on MAIT cells from multiple NHP species (21, 25, 27, 28).

## Distribution of Mucosal-Associated Invariant T-Cells in Non-Human Primates

MAIT cells constitute approximately 2.6% (range: 0.1–9.2%) of the total T-cell population in human peripheral blood (Table 1) (40). By comparison, MAIT cell frequencies in the peripheral blood range from 0.3 to 4.8% in RMs (21, 27), 0.026–1.28% in PTM (25), and 0.6–17% of CD3^+^CD8^+^ T-cells in MCMs (28). In general, NHPs maintain peripheral MAIT cell frequencies at 1–2% of the bulk CD3 T-cell population. While MAIT cell frequencies are lower than what is observed in humans, it is substantially higher than baseline MAIT cell frequencies in mice (16), and is sufficient to evaluate changes in peripheral MAIT cell frequencies and phenotype with different experimental interventions.

**TABLE 1:** Comparison of MAIT cell tissue frequencies between humans, non-human primates, and mice.

	Human (% of CD3^+^); Mean (range)	Non-human primate (% of CD3^+^); Mean (range)	Mouse (% of CD3^+^); Mean (range)
Blood	2.6 (0.1–9.2)	1–2 (0.026–6.5)	0.1
	(40)	(21, 25, 27–29)	(16)
Liver	20–50	4–7 (0.5–17)	0.60
	(41)	(21, 23, 25–27)	(16)
Gastrointestinal tract	1.5–10	0.02–3	0.70
	(41)	(21, 23, 25–27)	(16)
Lung	2–4	1.10	3.30
	(41)	(27)	(16)
Lymph node	1	0.03–0.2	0.20
	(38, 45)	(21, 25)	(16)

Similar to MAIT cell frequencies in the blood, NHPs MAIT cell frequencies in tissues tend to be lower compared to the frequencies in humans, but higher compared to murine MAIT cell frequencies. Both humans and NHPs maintain relatively higher MAIT cells frequencies in the liver and mucosal tissue sites (e.g., Gastrointestinal tract, lung), compared to their respective peripheral blood frequencies. MAIT cell frequencies in the human liver vary from 20 to 50% of the bulk CD3^+^ T-cell population [reviewed in Kurioka et al. (41)], and are significantly enriched in the liver of PTM (approximately 5% of CD3^+^ T-cell population) compared to paired PBMCs (approximately 1% of CD3^+^ T-cell population) (25). MAIT cells comprise a range of ∼0.5–9.8% of the bulk CD3^+^ T-cell population in the liver of RMs (21, 23, 26, 27). Within the gastrointestinal tract, MAIT cell frequencies in humans vary from 1.5% of the bulk T-cell population to approximately 60% of the CD3^+^CD8^+^ population, with the highest concentration of MAIT cells occurring in the jejunum [(19), reviewed in Kurioka et al. (41)]. MAIT cell frequencies range from 0.02 to 0.03% of CD3^+^ T-cells in the rectal mucosa of PTM (25). However, there was no significant MAIT cell enrichment in the rectal mucosa compared to paired PBMC’s (0.15–1.31% of CD3^+^ T-cell population). This was attributed to a relatively low expression of the mucosal homing marker α4β7 in circulating MAIT cells in PTM. MAIT cells comprised ∼1–3% of the bulk CD3^+^T-cell population in the gastrointestinal tract of RMs (23, 26, 27) with one study noting the highest frequency occurring in the colon compared to the ileum and jejunum (23).

MAIT cells are relatively abundant in the human lung, accounting for approximately 2–4% of the bulk T-cell population, with the most dramatic enrichment occurring in the trachea, and the proximal and distal bronchi [(42), reviewed in Kurioka et al. (41)]. By comparison, MAIT cells account for approximately 1.1% of the bulk T-cell population, or 16% (range: 9–28%) of the bulk CD3^+^CD8^+^ T-cell population, in the lungs of RMs (22, 27). Additionally, MAIT cell frequencies are highly variable in RM bronchoalveolar lavage fluid (BAL), comprising 0.5–14.7% of the bulk CD3^+^ T-cell population (21, 23, 24, 26), or approximately 1–17% of the bulk CD3^+^CD8^+^ T-cell population (29). Similar findings have also been made in MCMs, with MAIT cell frequencies ranging from approximately 12–40% of the bulk CD3^+^CD8^+^ T-cell population in the BAL (28).

MAIT cells are consistently maintained at low frequencies in secondary lymphoid organs (lymph nodes and spleen) compared to the peripheral blood, in both humans and NHPs. This is attributed to the relative lack of CCR7 and CD62L expression, both required for lymphoid tissue homing, on peripheral MAIT cells [reviewed in Kurioka et al. (41)]. Additionally, it has been postulated that MAIT cells in the lymph represent a distinct population that recirculate from the tissues, separate from circulating MAIT cells in the blood (20). While similar patterns of tissue recirculation in NHPs still need to be investigated, differential distribution of MAIT cell phenotypes between lymphoid and extralymphoid tissues has been described in RMs. MAIT cells within secondary lymphoid tissues are predominately CCR7^+^CD28^+^ (central memory), while MAIT cells in extralymphoid tissues are predominately CCR7^–^CD28^+^ (transitional memory) or CCR7^–^CD28^–^ (effector memory) (23).

## Functional Activity of Non-Human Primates Mucosal-Associated Invariant T-Cells

**FIGURE 1:**
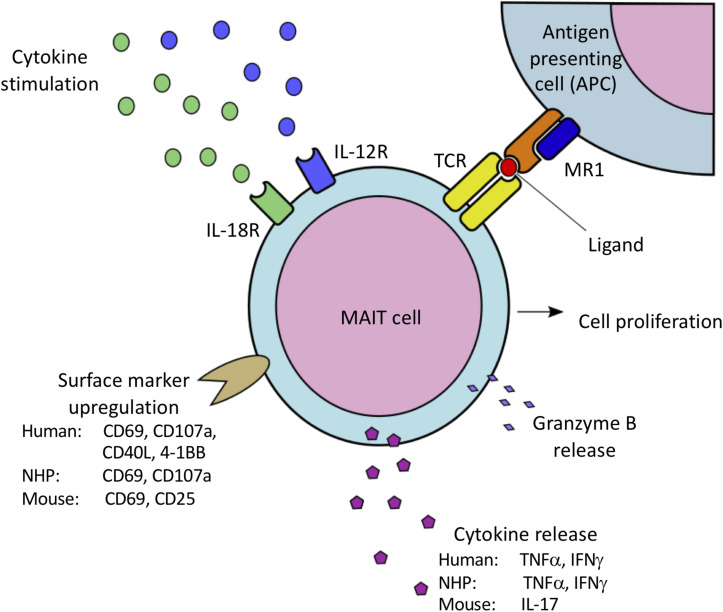
Species-specific variations in MAIT cell activation via MR1-mediated presentation of bacterial-derived vitamin B metabolites to the MAIT cell TCR, as well as IL-12/IL-18 mediated cytokine stimulation. Activation results in MAIT cell proliferation, cell surface marker upregulation associated with cell activation, and release of proinflammatory cytokines and granzyme B. Both human and NHP MAIT cells upregulate CD69 and CD107a, and produce IFNγ and TNFα. Human MAIT cells have also been shown to upregulate CD40L and 4-1BB. In contrast, murine MAIT cells have been shown to upregulate CD69 and CD25, and predominately produce IL-17 when activated.

Species specific variations in T-cell functional activity are important to consider in the context of different animal models. Stimulation of peripheral blood mononuclear cells (PBMCs) from PTM with the riboflavin metabolite–based antigen 5-OP-RU results in a dose-dependent upregulation of CD69 and selective proliferation of MR1 tetramer^+^Vα7.2^+^ MAIT cells (Table 2) (25), demonstrating that human and macaque MAIT cells are similarly activated by antigen (Figure 1). Surprisingly, 5-OP-RU stimulated PTM MAIT cells produced relatively low amounts of TNFα and IFNγ (although IFNγ secretion could be augmented with IL-12 and IL-18 supplementation to the cultures). In contrast to the PTM data, stimulation of human peripheral MAIT cells with 5-A-RU resulted in significant production of TNFα and IFNγ (43). In contrast to humans and NHPs, murine MAIT cells predominately produce IL-17 following stimulation with 5-OP-RU (16), highlighting the potential for species specific differences in MAIT cells functional activity, and the value of the NHPs in being able to better model the human peripheral MAIT cell cytokine profile.

**TABLE 2:** Comparison of MAIT stimulation responses between humans, non-human primates, and mice.

	Human (% of CD3^+^); Mean (range)	Non-human primate (% of CD3^+^); Mean (range)
5-OP-RU/5-A-RU	CD69: ∼95 TNFα: 14–18 IFNγ: 20–60	CD69: 20–80 TNFα: 5–13 IFNγ: 0.1–4
	(43, 59)	(25)
PMA/Ionomycin	TNFα: ∼80–95 IFNγ: ∼0.5–5 IL-17: ∼4–10	TNFα: ∼20–80 IFNγ: ∼5–80 IL-17: ∼7–20
	(45, 60)	(25, 27)
*Fixed E. coli*	TNFα: 10–50 IFNγ: 10–75	TNFα: ∼25–35 IFNγ: ∼20–35
	(43, 61)	(28)

The use of fixed *E. coli* to stimulate human MAIT cells is now a standard assay, and activates MAIT cells through both MR1-dependent and -independent mechanisms (6, 44). Similarly, LPS or *E. coli* stimulation resulted in significant IFNγ and TNFα production in RM and MCM MAIT cells (23, 28), as well as CD107a upregulation in MCM MAIT cells (28). In contrast, MCM MAIT cell stimulation with *Mycobacterium smegmatis* resulted in no significant increase in IFNγ and TNFα production, or CD107a upregulation.

Following mitogenic stimulation with PMA/ionomycin, both PTM and RM MAIT cells readily produce IFNγ, TNFα, and IL-17 (21, 25, 27). Variations in the amount of IL-17 production have been reported, with PTM MAIT cells producing relatively moderate levels of IL-17, and RM MAIT cells producing higher levels of IL-17. These differences may reflect species specific variability in MAIT cell IL-17 production capacity. These findings are similar to PMA/ionomycin stimulated human MAIT cells, which results in IFNγ, TNFα, and IL-17 production (45). Compared to humans, NHP MAIT cells have fairly conserved functional capacities, further highlighting the utility of NHP model to study immune dynamics.

## Mucosal-Associated Invariant T-Cells in Acute SIV/SHIV Infection

HIV infection in humans is marked by significant immune dysregulation, with significant MAIT cell depletion occurring in the peripheral circulation within the first 4 months of infection (33). However, within the first weeks of infection, longitudinal data suggests that there is a transient increase in MAIT cell frequencies in the peripheral circulation (46). Consistent with this observed MAIT cell expansion during acute HIV infection, NHP MAIT cells also appear to be activated and/or proliferating during the first month post-SIV/SHIV infection. Two studies have evaluated longitudinal MAIT cell dynamics in macaques following SIV/SHIV infection (25), and both noted increased expression of the proliferation marker, Ki-67, in circulating MAIT cells during acute SIV/SHIV infection. Increases in peripheral MAIT cell frequency were only observed in one of the two studies (25), but large fluctuations in pre-infection MAIT cell frequencies, high inter-animal variability in MAIT cell frequency, and small sample size, may have contributed to the lack of observed trends in the second study (28). MAIT cell frequencies also increase in the rectal mucosa and lymph nodes following SHIV challenge, with a peak frequency at ∼3 weeks post-infection (25). These results highlight the conservation of blood and tissue MAIT cell frequencies between humans during acute HIV infection, and NHPs following SIV/SHIV infection.

Evaluation of activation and tissue trafficking markers in circulating MAIT cells have shown variable changes following acute SIV/SHIV infection. There is no change in CCR5, CCR6, CXCR3, CD69, and HLA-DR expression on PTM peripheral blood MAIT cells during acute SHIV infection (25). Similarly, there was no change in CD69 expression on the effector memory (CD28^–^CD95^+^) MAIT cell population of MCMs following SIV challenge (28). However, a transient but significant increase in CD69, CD39, T-bet, and RORγT was observed in the central memory (CD28^+^CD95^+^) MAIT cell population of MCMs at 2–3 weeks post-SIV challenge (28).

SIV/SHIV infection is associated with a transient increase inperipheral blood α4β7^+^ MAIT cells in PTMs at 3–4 weeks post infection (25). This increase correlated with peak viral load, as well as increased MAIT cell frequencies with rectal mucosa. One hypothesis is that early viral replication and increased antigen availability in the lymph node may drive α4β7 expression on MAIT cells. Peripheral blood MAIT cells upregulated α4β7 when cocultured with mesenteric lymph node cells and 5-OP-RU, demonstrating that circulating MAIT cells can upregulate α4β7 when stimulated with antigen in the context of mesenteric lymph node cells (25). These findings are similar to what has been observed with conventional T-cells in humans and mice, where α4β7 induction is mediated by retinoic acid-producing dendritic cells from gut-associated lymphoid tissues and mesenteric lymph nodes (46–50). Similarly, α4β7 expression is significantly increased on peripheral MAIT cells from chronic SHIV-infected RMs, compared to naïve controls (27). In humans, β7 integrin expression is increased in HIV^+^ individuals, suggesting this relationship may be conserved between humans and NHP models [reviewed in Saeidi et al. (51)]. This may also partially explain the mechanism for the transient increase in MAIT cell frequencies observed in the rectal mucosa of HIV^+^ individuals during initial infection (46). Alternatively, increased α4β7^+^ MAIT cells in the peripheral blood following SIV/SHIV infection may be due to expansion and subsequent trafficking of MAIT cells into the blood from mucosal tissues sites. These changes correlate with peak viremia, suggesting that early viral replication may drive α4β7 induction and increase mucosal tissue trafficking during acute infection. This is potentially driven by direct viral replication, or immune dysregulation with microbial translocation in the gastrointestinal tract. The lack of MAIT cell depletion during acute infection is consistent with what has been observed in humans during initial HIV infection and emphasizes the importance of the NHP-SIV/SHIV model to study MAIT cell dynamics during peracute infection.

## Mucosal-Associated Invariant T-Cells in Chronic SIV/SHIV Infection

Chronic HIV-1 infection in humans is associated with persistent MAIT cell depletion in the peripheral circulation [(33, 37), reviewed in Saeidi et al. and Juno et al. (51, 52)]. In contrast, chronic SIV/SHIV infection has inconsistent effects on peripheral MAIT cell frequencies in NHPs. One study reported a reduced frequency and absolute number of peripheral MAIT cells in RMs during chronic SIV infection (21). A reduction in CCR6^+^ MAIT cells was also noted during chronic SIV infection, which was hypothesized to reflect impaired tissue trafficking capabilities of the remaining MAIT cells in the periphery. In contrast, a second study noted increased peripheral MAIT cell frequencies in RMs during chronic SHIV infection, which correlated with plasma viral load (27). Despite the overall increase in frequency, MAIT cell frequencies in the peripheral blood were inversely correlated with plasma viral load, and directly correlated with peripheral CD4^+^ T-cell frequencies. Human MAIT cells are not preferentially infected by HIV (53), suggesting that the observed correlation may reflect reduced MAIT cell frequencies due to reduced CD4 T-cell frequencies, which is secondary to virus-mediated CD4 T-cell depletion. Other potential causes for the observed correlation includes increased MAIT cell consumption in response to higher amounts of virus mediated gastrointestinal inflammation and bacterial translocation. A third study identified no significant difference in peripheral MAIT cell frequencies during chronic SHIV infection in PTMs (25). Increased Ki-67 expression in the peripheral MAIT cell populations was identified in 2 studies, despite the variability in MAIT cell frequency (21, 27).

Similar variability has also been observed in MAIT cell frequencies in tissues during chronic SIV/SHIV infection in NHPs. Two separate studies have reported increased and decreased MAIT cell frequencies in the BAL fluid of chronic-SHIV infected PTMs and chronic-SIV infected RMs, respectively (21, 25). Additionally, one study identified decreased MAIT cell frequencies in the inguinal lymph nodes, with concurrent maintenance of MAIT cells in the mesenteric lymph nodes of chronic SHIV-infected PTMs (25). In contrast, a separate study identified a reduction in MAIT cell frequencies in the mesenteric lymph nodes of chronic SIV-infected RMs (21). However, MAIT cell frequencies are consistently maintained in the liver, spleen, and gastrointestinal tract of chronic SIV/SHIV-infected macaques (21, 25).

Chronic HIV infection is associated with altered expression of a variety of cytokine and chemokine receptors on peripheral MAIT cells [reviewed in Saeidi et al. (51)]. Similar alterations have also been observed in chronic SIV/SHIV infected NHPs. Chronic SHIV-infection is associated with reduced Tbet and Eomes expression of peripheral MAIT cells in PTMs, while CCR5 and CXCR3 expression levels are unchanged (25). In contrast to humans where CCR6 and HLA-DR expression is decreased and increased, respectively in MAIT cells from chronic HIV-infected individuals, CCR6 and HLA-DR expression on peripheral MAIT cells are unchanged in chronic SHIV-infected PTMs. Chronic SIV infection in RMs results in reduced CD28 and PLZF expression on peripheral MAIT cells, while caspase 3 expression is unchanged (21). Reduced Tbet and Eomes expression in both humans and NHPs during chronic viral infections indicates some conservation of the immune dysregulation within the model. However, the NHP SIV/SHIV model likely does not capture every aspect of the immune dysregulation that is observed in HIV patients. Some examples where immune dysregulation varies includes MAIT cell activation (HLA-DR), antigen presentation (HLA-DR), and tissue trafficking (CCR6). Further studies are needed to better characterize MAIT cell functional and phenotypic changes that occur during SIV/SHIV infection.

Immune effector functions of peripheral MAIT cells are significantly impaired during chronic HIV infection. This is marked by reduced cytokine production and CD69 upregulation following *E. coli* stimulation, which is only partially restored with combination antiretroviral therapy [(37), reviewed in Saeidi et al. (51)]. Similar findings were also reported in one study evaluating chronic SHIV-infected macaques, with reduced TNFα and IL-17 production capacity following mitogenic stimulation (27). However, two studies looking at chronic SIV and SHIV infected MCMs and PTMs, respectively, report no alterations in cytokine stimulation capacity (25, 28). Additionally, chronic SHIV-infection in PTMs is associated with increased CD69 expression on peripheral MAIT cells (25). SIV/SHIV infection does not consistently alter MAIT cells’ ability to produce granzyme, with 2 studies noting no significant changes in the frequency of granzyme B^+^ MAIT cells in the peripheral blood during SIV or SHIV infection (27, 29). These findings are in contrast to what is observed in HIV^+^ individuals, where peripheral granzyme B^+^ MAIT cells are increased in frequency compared to uninfected control samples at resting state (54) and have reduced perforin and granzyme expression following *E. coli* stimulation (54, 55). In one of the NHP studies, perforin^+^ and perforin^+^granzyme B^+^ MAIT cell frequencies were elevated following SHIV challenge (27).

The cause for the variability in MAIT cell frequencies, receptor expression, and effector functions observed in these studies is unknown. The authors hypothesize these may reflect species differences between RMs and PTMs, and/or differences in viral pathogenesis between different SIV/SHIV strains. Additional sources of variation may be due to inter-study differences in gating strategies to identify MAIT cells, including the usage of CD161 expression and CD8^+^ pre-gating. There is also evidence of significant variability in MAIT cell frequencies within the peripheral circulation and different tissue compartments, both between different species and individual animals. This emphasizes the need for longitudinal studies, in order to evaluate changes in MAIT cell trends following different experimental manipulations, as opposed to cross sectional studies. It will also be important to consider standards for identifying NHP MAIT cells in future studies, including the usage of the Vα7.2 TCR and species specific MR1 tetramer reactivity, due to evidence of limited cross-reactivity with human MR1 tetramers.

## Mucosal-Associated Invariant T-Cells in Chronic SIV/*Mtb* Co-Infection

One important consideration for HIV^+^ individuals is the risk of co-infection with secondary opportunistic bacterial and fungal pathogens, for which HIV-mediated dysregulation of MAIT cells may be a predisposing factor. While data on the impact of MAIT cell responses on fungal infections is currently lacking, MAIT cells have been shown to become activated in response to *Candida* spp. [reviewed in Wong et al. (56)] and *Aspergillus* spp. (57). *Mycobacterium tuberculosis* (*Mtb*) is one of the most common opportunistic bacterial infection among HIV^+^ individuals. Individuals with active or latent *Mtb* infections tend to have lower peripheral MAIT cell frequencies, compared to uninfected individuals (58). Additionally, MAIT cells from HIV/*Mtb* co-infected individuals tended to express lower levels of CCR6 compared to HIV infected individuals and healthy controls, though statistically significant differences were only seen between the HIV/*Mtb* co-infected individuals and the healthy controls. This suggests that HIV-mediated MAIT cell dysregulation may predispose individuals to opportunistic infection with *Mtb*.

In RMs, vaccination with Bacillus Calmette–Guérin, a vaccine historically used to prevent tuberculosis, resulted in significantly increased expression of Ki-67 and granzyme B in MAIT cells in the peripheral blood, the vaccine sites, and the draining lymph nodes, compared to pre-vaccination frequencies (23). While there was evidence of significant MAIT cell activation following vaccination, no significant changes in MAIT cell frequencies in the blood or tissue sites were observed. Increased peripheral MAIT cell Ki-67 expression, without an increased in peripheral blood MAIT cell frequencies, was also observed in RMs following intrabronchial *Mtb* infection (23). No significant difference in MAIT cell frequencies in the peripheral blood or tissue has been reported with SIV/*Mtb* co-infection, similar to what has been observed in humans (26, 28, 58). However, a weak negative correlation between MAIT cell frequency and bacterial burden was observed in SIV/*Mtb* co-infected MCMs, which was not maintained in NHPs mono-infected with *Mtb*. There was also a significant reduction in TNFα producing capacity of MAIT cells from the peripheral blood and *Mtb* granulomas of SIV/*Mtb* co-infected MCMs, compared to *Mtb* mono-infected controls (28). The results reflect the potential impact of MAIT cells on the susceptibility of *Mtb* in HIV^+^ individuals, in an NHP animal model.

## Conclusion and Future Directions

There is growing evidence that MAIT cells are capable of being activated in response to viral pathogens, despite the lack of vitamin B metabolites that are necessary for TCR-dependent MAIT cell activation. Additionally, there is evidence of altered MAIT cell frequencies, phenotypes, and functional activity during both acute and chronic HIV infection. However, the underlying pathophysiology for why these changes occur is not well understood. Having a better understanding of this interaction may provide key insights into developing vaccines and/or therapies for HIV infection and related co-morbidities. The NHP animal model provides a valuable platform to study these changes, and understand the underlying mechanisms for why they occur.

While SIV/SHIV infection in NHPs does not reliably recapitulate all aspects of the MAIT cell changes that occur during HIV infection, notably the lack of MAIT cell depletion, it does model many aspects of HIV infection well. This includes an initial increase in MAIT cell frequency with upregulation of α4β7 during acute viral replication, as well as evidence of impaired MAIT cell function in SIV/mTB co-infected NHPs. Future studies should focus on characterizing the perturbations in the MAIT cell populations within different tissue compartments during SIV/SHIV infection; with a special focus on variables that may alter these perturbations, such as macaque species, age, and gender, as well as strain, dose, and route of administration of the virus. By better understanding how these factors impact MAIT cell dynamics during SIV/SHIV infection, we will ideally be able to refine the NHP model to better recapitulate the MAIT cell changes that occur during HIV infection in humans. Additionally, the robust response that NHP MAIT cells generate to both antigen and cytokines, including cytokine independent cell activation, which is comparable to humans, may be advantageous for studying MAIT cell dynamics with other pathogens.

## Author Contributions

IB-A wrote the initial draft of the manuscript. IB-A, SK, and JJ revised the manuscript. All authors contributed to the article and approved the submitted version.

## Conflict of Interest

The authors declare that the research was conducted in the absence of any commercial or financial relationships that could be construed as a potential conflict of interest.
